# On Functional Disease of the Liver, Associated with Uterine Derangement

**Published:** 1849-12

**Authors:** Butler Lane


### SELECTIONS

FROM

### AMERICAN AND FOREIGN JOURNALS.

---

*On Functional Disease of the Liver, associated with Uterine Derangement.* By Dr. Butler Lane.—[Dr. Lane thinks that the relations between the liver and the uterus in health and disease, have not been sufficiently noticed. He says :]

The research and experience of three years enabled me to advance the following propositions :—

1. In a state of health an anatomical and physiological relationship exists in the female between the liver and the uterus.

2. The relationship in question is apt to be disturbed in many forms of disorder and disease, primarily implicating either organ individually.

3. The disturbance in question, though varying much in nature and degree, assumes certain definite aspects, the recognition of which serves much to direct and facilitate our therapeutic appliances.

In a great majority of instances it will be found that menstruation rarely occurs without some concomitant alvine derangement. The bowels will vary from their usual state of action, becoming either confined or relaxed relatively to their ordinary condition. Now we know that bile may be considered as the natural stimulus to healthy alvine action ; if therefore, that secretion be increased or diminished, so in proportion will there consequently be increase or diminution in the action of the bowels. Assuming, therefore, the existence of an hepatico-uterine relation, it will follow that when the periodical excitement of the uterus takes place, occasioning menstruation, then the liver will manifest some sympathetic influence. If the action should be simply derivative, the biliary secretion will be diminished, and the natural catharsis will consequently be lessened. But if a greater degree of excitement attend the menstrual period, the liver may be even supposed to undergo a sympathetic degree of excitement or erethism, and its secretion being thereby augmented, instead of being diminished, increased catharsis will consequently result. It is also to be remembered that there is an immediate connection between the veins of the uterus and the portal system, which must exert some influence. The uterine blood, however, when subjected to its own special secretive action, is purified by that very process, and consequently it does not in that respect need the hepatic elaboration; it is unlike the intestinal blood, which is impoverished by furnishing pabulum for the secretions and deteriorated by the admixture of foreign matter imbibed by venous absorption.

In some instances, certainly, intestinal irritation will cause uterine excitement, and on the other hand, I can conceive that uterine excitement may implicate the intestinal canal; but I cannot understand how uterine excitement is, as a general rule, to diminish the normal irritability of the intestines, and the cathartic action thereon contingent, unless it be through the medium of hepatic influence. It seems at first sight rather irrational to consider the secretion from the portal venous system as antagonized by that from the arterial uterine system; but we must remember that, during the menstrual period, a complete temporary revolution takes place in the female constitution, which, indeed, is often very perceptible at its outset. In such cases langour and oppression are experienced, the pulse is accelerated, the countenance becomes sallow and almost jaundiced, a peculiar faint odor emanates with the breath and from the cutaneous surface, and only on the establishment of the uterine flux do these symptoms subside. I believe that at the approach of the uterine excitement the function of the liver is performed imperfectly, the process of depuration therein effected is incomplete, and the supplementary respiratory action, though often much excited, is inadequate to the task.—Thus the arterial blood circulating through the system is to some extent impure ; this very impurity is the essence of the peculiar constitutional state that exists, and it is by means of the uterine secretion that the blood undergoes the necessary process of filtration and separation, by which the system becomes relieved. Chlorosis has often been regarded as an hepatic affection, but it is only by adopting the above views that the assumption admits of explanation.—*Med. Gaz.*

